# Fatty Acids and Their Lipogenic Enzymes in Anorexia Nervosa Clinical Subtypes

**DOI:** 10.3390/ijms25105516

**Published:** 2024-05-18

**Authors:** Nhien Nguyen, D. Blake Woodside, Eileen Lam, Oswald Quehenberger, J. Bruce German, Pei-an Betty Shih

**Affiliations:** 1 Department of Psychiatry, University of California, San Diego, La Jolla, CA 92037, USA; 2 Centre for Mental Health, University Health Network, Toronto, ON M5G 2C4, Canada; 3 Department of Pharmacology, University of California, San Diego, La Jolla, CA 92093, USA; 4 Department of Food Science & Technology, University of California, Davis, Davis, CA 95616, USA; jbgerman@ucdavis.edu

**Keywords:** fatty acids, desaturases, elongases, anorexia nervosa

## Abstract

Disordered eating behavior differs between the restricting subtype (AN-R) and the binging and purging subtype (AN-BP) of anorexia nervosa (AN). Yet, little is known about how these differences impact fatty acid (FA) dysregulation in AN. To address this question, we analyzed 26 FAs and 7 FA lipogenic enzymes (4 desaturases and 3 elongases) in 96 women: 25 AN-R, 25 AN-BP, and 46 healthy control women. Our goal was to assess subtype-specific patterns. Lauric acid was significantly higher in AN-BP than in AN-R at the fasting timepoint (*p* = 0.038) and displayed significantly different postprandial changes 2 h after eating. AN-R displayed significantly higher levels of n-3 alpha-linolenic acid, stearidonic acid, eicosapentaenoic acid (EPA), docosapentaenoic acid, and n-6 linoleic acid and gamma-linolenic acid compared to controls. AN-BP showed elevated EPA and saturated lauric acid compared to controls. Higher EPA was associated with elevated anxiety in AN-R (*p* = 0.035) but was linked to lower anxiety in AN-BP (*p* = 0.043). These findings suggest distinct disordered eating behaviors in AN subtypes contribute to lipid dysregulation and eating disorder comorbidities. A personalized dietary intervention may improve lipid dysregulation and enhance treatment effectiveness for AN.

## 1. Introduction

Anorexia nervosa (AN) is an eating disorder that affects up to 4% of women and 0.3% of men [1]. The mortality rates for AN range from 4.4% to 18%, [2,3], making it one of the deadliest psychiatric disorders. AN patients display a disturbed body image perception, profound fear of weight gain, and extreme dietary restrictions and/or maladaptive eating behaviors that result in excessive weight loss and low BMI [4]. AN patients are known to be treatment-resistant [5] and have shown high relapse rates ranging from 9% to 42% [6,7,8]. Avoidance of high-calorie, high-fat foods is a well-characterized behavior underlying pathological eating in AN [9,10,11,12]. AN patients consume significantly fewer calories and less fat even after weight recovery [13,14,15,16]. Low dietary fat was linked to longer illness duration [17] and unsatisfactory treatment outcomes such as relapse [18,19], highlighting the need to understand the role lipids play in AN.

High-fat foods that AN patients typically refuse to consume are major dietary sources of saturated, monounsaturated, and polyunsaturated (n-3, n-6, n-7, n-9) fatty acids (FAs). We and others have reported data to support the presence of lipid dysregulation in AN, including increased saturated, n-3, n-7, and n-9 FAs [20,21,22,23,24,25] and reduced total n-6 polyunsaturated FA (PUFA) [20,21,22,23,24] compared to healthy controls. N-3 PUFAs are considered “beneficial lipids” and are often recommended for the general population to maintain health [26]. While the increased n-3 PUFAs in AN may have resulted from relatively higher intakes of “healthy foods” with high n-3 PUFA, such as vegetables and fish [25], endogenous factors such as metabolism and gut microbiota may also play a role. Human genetics and genomics studies have highlighted the involvement of metabolic dysregulation in AN pathogenesis [27,28,29]. Thus, it is important to understand how lipids and metabolic dysregulation affect AN risk and psychopathology.

AN is classified into the restricting subtype (AN-R) and the binging and purging subtypes (AN-BP) in the Diagnostic and Statistical Manual of Mental Disorders (DSM-5) [30] based on the subtype’s pathological eating behaviors. Although the two subtypes can cross over throughout a patient’s lifespan with crossover rates ranging from 17% to 64% [7,31], significant differences in subtypes’ prognosis and outcomes have been well documented. Compared to AN-R, AN-BP patients have shown higher rates of mortality [32,33] and relapse [34], more severe psychopathology [35,36,37] and psychiatric comorbidities [38,39,40], and poorer treatment response and compliance rates [34,41,42,43,44,45]. These differences in clinical outcomes between subtypes may be linked to their distinct disordered eating behaviors.

AN-R individuals regularly fast and restrict food intake [30], while AN-BP individuals binge-eat in a short time window, then purge/vomit what they eat to lose weight [30]. AN-BP consume more starchy foods, fewer dairy products [12], have a more irregular meal schedule [46], and display more emotional eating [47,48] compared to AN-R. These dietary behavioral differences can lead to significantly different FA concentrations and compositions in vivo. Yet, with the exception of one inpatient treatment study from Japan [22], no other report has documented and compared lipid levels and composition between the two AN subtypes. Moreover, the disparity in subtypes’ clinical trajectory may be directly linked to their distinct disordered eating behaviors and FA dysregulation, emphasizing the need to stratify lipids investigation by AN subtypes.

Given the complex relationships among eating behavior, diet, and lipid metabolism, as well as their synergistic effects on human health, we leveraged the known eating behavioral differences between the two AN subtypes to clarify how disordered eating behavior impacts lipid dysregulation in AN. We applied a standardized meal challenge protocol to examine AN subtype-stratified associations between FAs and AN psychopathology. Our results revealed significantly different meal-associated lipid changes in AN compared to healthy controls and showed distinct PUFA associations with anxiety and depression in the two AN subtypes. The knowledge obtained from this investigation is needed to better understand the unique lipid dysregulation present in each AN subtype and how the dysregulated FAs link to AN risk and psychopathology. Disordered eating behavior and dietary intake are modifiable; therefore, our results can guide the development of personalized interventions to normalize disordered eating and aberrant lipid metabolism, leading to improved treatment compliance and recovery.

## 2. Results

### 2.1. Characteristics of Study Participants

This study included 50 women with AN (age: 30.18 ± 10.19 years) and 46 healthy control women (age: 30.67 ± 9.33 years). Of the 50 women with AN, 25 (50%) were classified as the restricting subtype (AN-R, age: 27.56 ± 8.18 years), and 25 (50%) were classified as the binging and purging subtype (AN-BP, age: 32.80 ± 11.44 years). A total of 30 (60%) of the 50 AN were ill AN (IAN), while 20 (40%) were weight-recovered AN (RecAN). 

Compared to controls, all AN had significantly lower BMI, higher depression, elevated fasting anxiety, higher fat food aversion, and a greater postprandial decrease in anxiety (ps < 0.001). Postprandial change in high-fat food aversion was directionally opposite and significantly different between all AN and controls (*p* = 0.03) (Table 1). 

Comparing the two AN subtypes, AN-BP were older (*p* = 0.07) and had a longer illness duration (*p* = 0.09). However, BMI, age of onset, depression, anxiety, food aversion, and postprandial changes in anxiety and food aversion did not statistically differ between the two subtypes (Table 1).

In all AN, 42 self-identified as White (84%), 7 as Asian (14%), and 1 as “more than one race” (2%). In controls, 26 self-identified as White (57%), 16 as Asian (35%), 2 as Black or African American (4%), and 2 as “more than one race” (4%). AN subjects had a higher proportion of Whites (*p* = 0.003) and a lower proportion of Asians (*p* = 0.02) compared to controls.

### 2.2. Elevated Levels of n-3- and n-6 PUFA in AN

**Figure 1:**
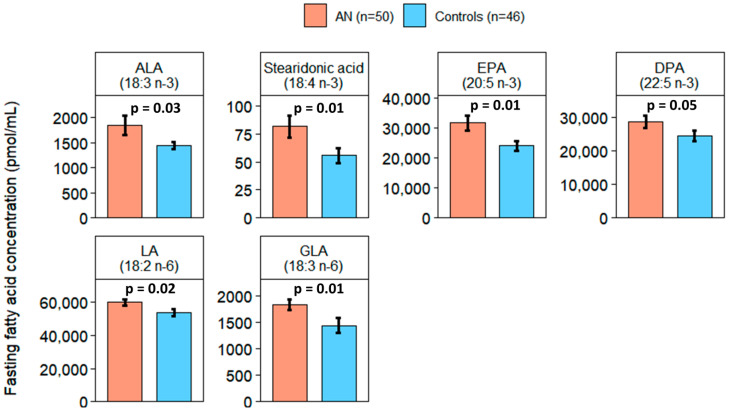
Fasting fatty acids significantly differed between AN (orange) and controls (blue). Bars and error bars represent covariate-adjusted mean and standard error of the mean, respectively (pmol/mL). *p* represents *p*-values from analysis of covariance (ANCOVA) models adjusted for age, BMI, and assay batch. ALA: alpha-linolenic acid; EPA: eicosapentaenoic acid; DPA: docosapentaenoic acid; LA: linoleic acid; GLA: gamma-linolenic acid.

**Figure 2:**
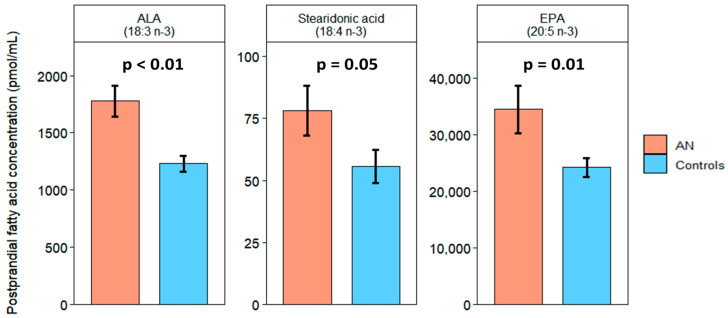
Postprandial fatty acids significantly differed between AN (orange) and controls (blue). Bars and error bars represent the covariate-adjusted mean and standard error of the mean, respectively (pmol/mL). *p* represents *p*-values from ANCOVA models adjusted for age, BMI, and assay batch. ALA: alpha-linolenic acid; EPA: eicosapentaenoic acid.

At the fasting timepoint, n-3 alpha-linolenic acid (ALA, 18:3: *p* = 0.03), stearidonic acid (18:4 n-3: *p* = 0.01), eicosapentaenoic acid (EPA, 20:5 n-3: *p* = 0.01), and docosapentaenoic acid (DPA, 22:5 n-3: *p* = 0.05) were higher in all AN compared to controls. N-6 linoleic acid (LA, 18:2: *p* = 0.016) and gamma-linoleic acid (GLA, 18:3 n-6: *p* = 0.01) were also higher in AN. (Figure 1). At the postprandial timepoint, ALA (*p* < 0.001), stearidonic acid (*p* = 0.05), and EPA (*p* = 0.01) remained higher in AN compared to controls (Figure 2). Total saturated FAs, monounsaturated FAs (MUFAs), PUFAs, and total n-3, n-6, n-7, and n-9 FAs did not statistically differ between all AN and controls at either timepoint. The unsaturated to saturated FA ratio (total unsaturated FA [MUFA + PUFA]/total saturated FA), polyunsaturated to saturated FA ratio (total PUFA/total saturated FA), and total n-6 to n-3 PUFA ratio (n-6/total n-3 PUFA) were not significantly different between AN and controls.

### 2.3. Fatty Acid Associations in AN Subtypes 

**Figure 3:**
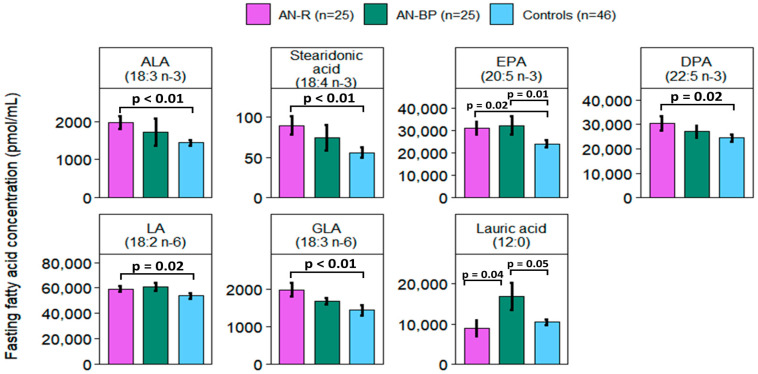
Fasting fatty acids significantly differed among restricting AN subtype (purple), binging and purging AN subtype (green), and controls (blue). Bars and error bars represent the mean and standard error of the mean (pmol/mL). *p* represents *p*-values from ANCOVA models adjusted for age, BMI, and assay batch. ALA and DPA were significantly different between AN-R and controls in pairwise comparisons but not in comparisons among the three groups. AN-R: restricting anorexia nervosa subtype; AN-BP: binging and purging anorexia nervosa subtype; ALA: alpha-linolenic acid; EPA: eicosapentaenoic acid; DPA: docosapentaenoic acid. LA: linoleic acid, GLA: gamma-linolenic acid.

At the fasting timepoint, saturated lauric acid (12:0) (*p* = 0.01), n-3 stearidonic acid (*p* = 0.04) and EPA (*p* = 0.02), and n-6 LA (*p* = 0.05) and GLA (*p* = 0.02) were significantly different among AN-R, AN-BP, and control groups (Figure 3). Among the two AN subtypes, lauric acid was higher in AN-BP than AN-R (*p* = 0.04), while other FAs showed no significant differences. EPA was elevated in both AN-BP (*p* = 0.01) and AN-R (*p* = 0.02) relative to controls. ALA (*p* < 0.01), stearidonic acid (*p* < 0.01), DPA (*p* = 0.02), LA (*p* = 0.03), and GLA (*p* < 0.01) were higher in AN-R, whereas lauric acid was higher in AN-BP compared to controls (*p* = 0.05) (Figure 3 and Table S1).

At the postprandial timepoint, ALA (*p* < 0.01) and EPA (*p* = 0.01) showed significant differences among AN-R, AN-BP, and control groups. None of the FAs differed statistically between the two AN subtypes. ALA was higher in both AN-R (*p* < 0.01) and AN-BP relative to controls (*p* = 0.01). Stearidonic acid (*p* = 0.03) was higher in AN-R, while EPA was higher in AN-BP compared to controls (*p* < 0.01). Total FA levels (saturated FAs, MUFAs, PUFAs, n-3, n-6, n-7, and n-9 FAs) and FA ratios (total unsaturated FA/total saturated FA, total PUFA/total saturated FA, and total n-6/total n-3 PUFA) did not differ among AN-R, AN-BP, and control groups at either timepoint (Table S2).

### 2.4. AN Recovery Status and Fatty Acids in AN Subtypes

AN recovery status was defined by weight and by the clinician’s assessment of eating disorder symptoms. Ill AN (IAN) had higher fasting n-3 stearidonic acid (81.72 ± 81.20 vs. 60.88 ± 46.12, *p* = 0.05), whereas n-3 docosahexaenoic acid was lower (DHA, 22:6: 121,602.44 ± 54,599.17 vs. 147,029.52 ± 71,075.88, *p* = 0.02) relative to controls. IAN also had higher n-6 GLA (1812.74 ± 293.04 vs. 1511.63 ± 942.19, *p* = 0.02) and total n-6/total n-3 PUFA ratio (2.97 ± 0.84 vs. 2.63 ± 0.78, *p* = 0.01) than in controls. In recovered AN (RecAN), n-3 ALA (1654.21 ± 612.98 vs. 1103.23 ± 410.56, *p* < 0.01), stearidonic acid (74.88 ± 30.11 vs. 50.66 ± 40.00, *p =* 0.02), EPA (33,046.55 ± 18,101.13 vs. 22,071.78 ± 10,759.35, *p* < 0.01), and n-6 LA (63,673.14 ± 14,151.49 vs. 55,728.63 ± 14,699.09, *p =* 0.05) were elevated compared to controls.

**Figure 4:**
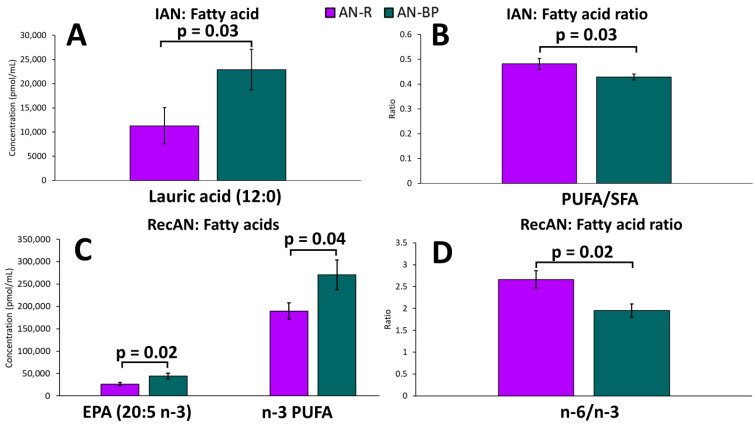
Fasting levels of fatty acids and fatty acid ratios that significantly differed between restricting AN (purple) and binging and purging AN (green) subtypes when comparing ill (**A**,**B**) and recovered states (**C**,**D**). Bars and error bars represent covariate-adjusted mean and standard error of the mean, respectively. *p* represents the *p*-value from ANCOVA models comparing the two AN subtypes when adjusted for age, BMI, and assay batch. IAN: ill anorexia nervosa; RecAN: recovered anorexia nervosa; AN-R: restricting AN; AN-BP: binging and purging AN; n-3 PUFA: total n-3 polyunsaturated fatty acids (ALA + stearidonic acid + 11,14,17-eicosatrienoic acid (ETE) + EPA + DPA + DHA); SFA: total saturated fatty acids (lauric acid + myristic acid + pentadecanoic acid + palmitic acid + margaric acid + stearic acid + arachidic acid).

Among AN patients only, none of the FAs differed between IAN and RecAN. The total n-6/total n-3 PUFA ratio was higher in IAN than in RecAN (2.73 ± 0.86 vs. 2.36 ± 0.71, *p* = 0.02). In IAN only, lauric acid was higher (*p* = 0.03), whereas the total PUFA/total saturated FA ratio was lower (*p* = 0.03) in AN-BP compared to AN-R (Figure 4). In RecAN (recovered for at least one year) only, EPA was higher in AN-BP compared to AN-R (*p* = 0.02). The total n-3 PUFA was higher (*p* = 0.04), while total n-6/total n-3 PUFA ratio was lower in AN-BP (*p* = 0.02) (Figure 4).

When we stratified by AN subtypes, in the AN-R subtype only, no significant differences were found in any FAs or FA ratios between ill and recovered AN-R. In the AN-BP subtype only, n-3 DHA (125,488.43 ± 63,446.41 vs. 175,049.39 ± 78,827.13 pmol/mL, *p* = 0.03) and saturated pentadecanoic acid (15:0: 9923.76 ± 4582.81 vs. 12,928.21 ± 4230.63 pmol/mL, *p* = 0.05) were lower, whereas the total n-6/total n-3 PUFA ratio was higher in ill AN-BP compared to recovered AN-BP (2.97 ± 0.91 vs. 2.14 ± 0.37, *p* < 0.01).

### 2.5. Lipogenic Enzyme Activity Indexes in AN and Controls

**Figure 5:**
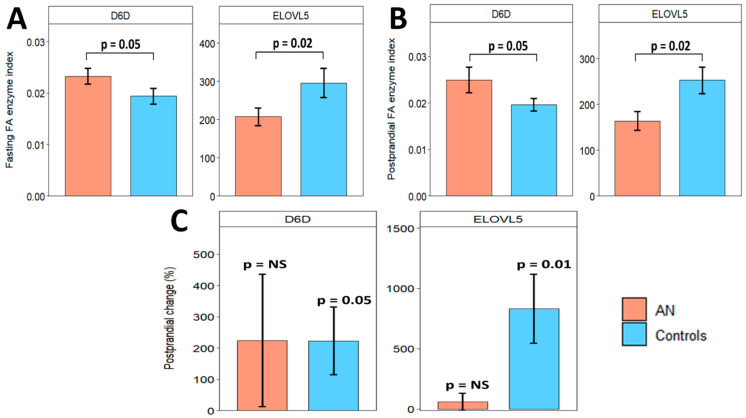
D6D and ELOVL5 were significantly different between all AN (orange) and controls (blue) at fasting (**A**) and postprandial (**B**) timepoints. Postprandial % changes in D6D and ELOVL5 within AN and control groups are shown in (**C**). Bars and error bars represent covariate-adjusted mean and standard error of the mean. *p* represents *p*-values from ANCOVA models adjusted for age, BMI, and assay batch. NS = non-significant (*p* ≥ 0.05). D6D: delta-6-desaturase (gamma-linolenic acid/linoleic acid); ELOVL5: elongase-5 (dihomo-gamma-linolenic acid/gamma-linolenic acid).

Lipogenic enzyme activity indexes (desaturase and elongase enzymes) were formulated using saturated FAs, MUFAs, and n-6 PUFAs. D6D and ELOVL5 significantly differed between all AN and controls at fasting and postprandial timepoints. At the fasting timepoint, D6D was higher (*p* = 0.05), whereas ELOVL5 was lower (*p* = 0.02) in all AN (Figure 5A). Two hours after eating, D6D remained higher (*p* = 0.05), while ELOVL5 remained lower in all ANs (*p* = 0.02) (Figure 5B).

At the fasting timepoint, D6D statistically differed among AN-R, AN-BP, and control groups (*p* = 0.04). Comparing each AN subtype to controls, D6D was higher (*p* = 0.01), whereas ELOVL5 was lower in AN-R than controls (*p* = 0.04). None of the enzymes differed between AN-BP and controls. Comparing the two AN subtypes, no significantly different markers were identified.

**Figure 6:**
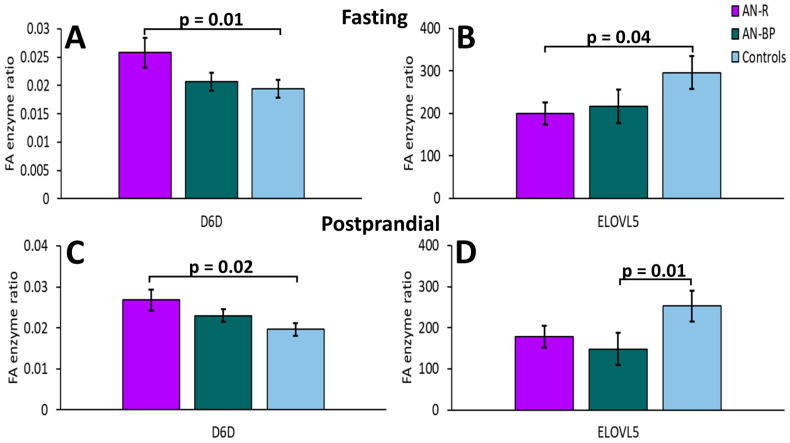
Enzyme activity indexes significantly differed between each AN subtype and the control group at fasting (**A**,**B**) and postprandial (**C**,**D**) timepoints. Bars and error bars represent covariate-adjusted mean and standard error of the mean, respectively. *p* represents *p*-value from ANCOVA model comparing each AN subtype to controls adjusted for age, BMI, and assay batch. D6D: delta-6-desaturase (gamma-linolenic acid/linoleic acid); ELOVL5: elongase-5 (dihomo-gamma-linolenic acid/gamma-linolenic acid); AN-R: restricting AN; AN-BP: binging and purging AN.

None of the enzymes differed significantly among the three groups or between the two subtypes at the postprandial timepoint. Comparing each AN subtype to controls, D6D was significantly higher in AN-R (*p* = 0.02), whereas ELOVL5 was significantly lower in AN-BP (*p* = 0.01) (Figure 6). 

### 2.6. Unexpected Differences in Postprandial Fatty Acids Changes between AN and Controls

Two hours after eating the study meal, none of the FAs significantly changed in all AN, whereas 15 FAs significantly increased in controls (Figure S1). In controls, n-3 ALA, stearidonic acid, EPA, DPA, and DHA increased by 199% (*p* = 0.01), 33% (*p* = 0.03), 13% (*p* = 0.02), 57% (*p* = 0.03), and 19% (*p* = 0.02), respectively. N-6 GLA, dihomo-gamma-linolenic acid (DGLA, 20:3 n-6), and arachidonic acid (ARA, 20:4 n-6) increased by 602% (*p* = 0.03), 17% (*p* = 0.04), and 16% (*p* = 0.03). N-9 mead acid (20:3), erucic acid (22:1 n-9), and nervonic acid (24:1 n-9) increased by 175% (*p* < 0.01), 43% (*p* = 0.01), and 86 (*p* = 0.02). Saturated myristic acid (14:0) and pentadecanoic acid (15:0) increased by 17% (*p* = 0.02) and 63% (*p* = 0.03), and both margaric acid (17:0) and stearic acid (18:0) increased by 28% (*p* = 0.03 and 0.04) (Figure S1). None of the enzymes significantly changed in all AN, whereas in controls, D6D and ELOVL5 increased by 223% (*p* = 0.05) and 830% (*p* = 0.01) (Figure 5C). 

After eating, saturated lauric acid increased by 64% in AN-R (*p* = 0.04) but decreased by 10% in AN-BP (*p* = 0.49), leading to a significant difference between the two subtypes (*p* = 0.04). Enzyme ELOVL2 decreased by 11% (*p* = 0.03) in AN-BP while increasing by 3% in AN-R (*p* = 0.60), resulting in a significant between-group difference (*p* = 0.04).

### 2.7. Associations of Fatty Acids and Enzymes with AN Phenotypes

#### 2.7.1. Association with BMI

**Figure 7:**
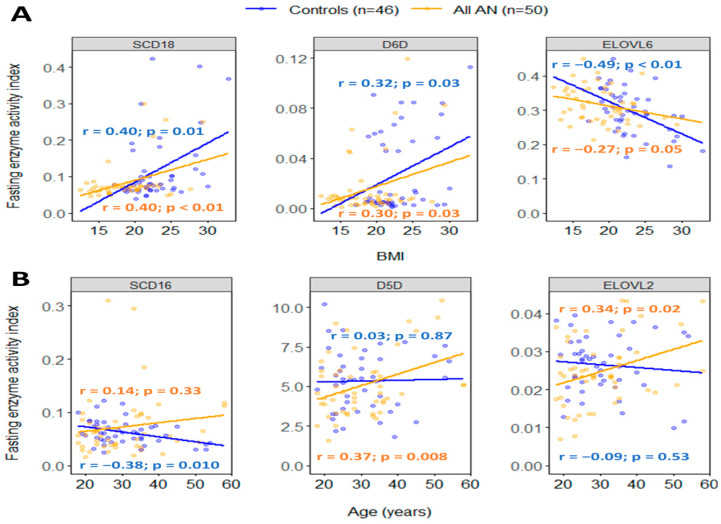
Significant Pearson’s correlations of BMI (**A**) and age (**B**) with fasting enzymes in all AN (orange) and controls (blue). Colored dots and solid lines represent individual data points and the slopes of relationships, respectively. r and *p* indicate Pearson’s correlation coefficient and *p*-value. BMI: body mass index (kg/m^2^); SCD16: stearoyl-CoA desaturase-16 (palmitoleic acid/palmitic acid); SCD18: stearoyl-CoA desaturase-18 (oleic acid/stearic acid); D6D: delta-6-desaturase (gamma-linolenic acid/linoleic acid); D5D: delta-5-desaturase (arachidonic acid/dihomo-gamma-linolenic acid); ELOVL6: elongase-6 (stearic acid/palmitic acid); ELOVL2: elongase-2 (adrenic acid/arachidonic acid).

Of n-3 PUFAs, ALA was inversely correlated with BMI in all AN (r = −0.37, *p* = 0.01) and controls (r = −0.34, *p* = 0.02). DPA was inversely correlated with BMI in AN only (r = −0.37, *p* = 0.01), whereas DHA was inversely correlated with BMI in controls only (r = −0.35, *p* = 0.02). N-6 GLA was positively correlated with BMI in all AN (r = 0.32, *p* = 0.02) and controls (r = 0.32, *p* = 0.03). N-9 erucic acid was inversely correlated with BMI in AN (r = −0.29, *p* = 0.05), while nervonic acid was inversely correlated with BMI in controls (r = −0.30, *p* = 0.04). Saturated stearic acid was inversely correlated with BMI in all AN (r = −0.31, *p* = 0.03) and controls (r = −0.36, *p* = 0.01), while lauric acid and margaric acid were inversely correlated with BMI in AN only (r = −0.31, *p* = 0.027 and r = −0.34, *p* = 0.02). None of the n-7 FAs significantly correlated with BMI in all AN or controls. Lipogenic enzymes SCD18 and D6D were positively correlated with BMI in both all AN (r = 0.40, *p* < 0.01 and r = 0.30, *p* = 0.03) and control groups (r = 0.04, *p* = 0.01 and r = 0.32, *p* = 0.03), while ELOVL6 was inversely correlated with BMI in controls only (r = −0.49, *p* < 0.01) (Figure 7A).

**Figure 8:**
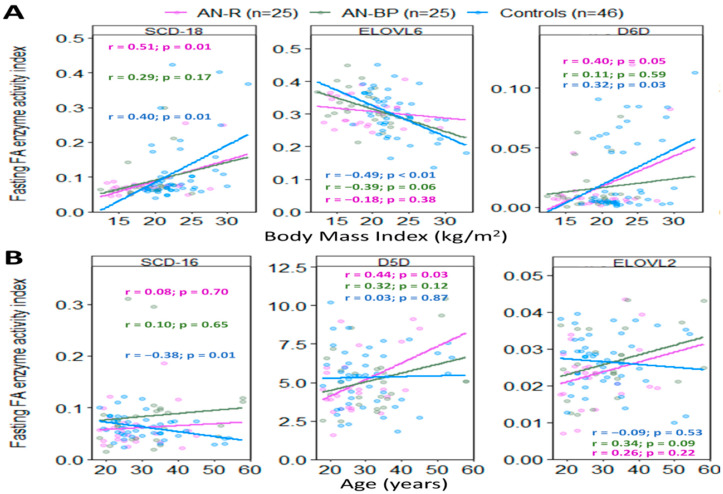
Pearson’s correlations of BMI (**A**) and age (**B**) with fasting enzymes in restricting AN subtype (purple), binging and purging AN subtype (green), and controls (blue). Colored dots and solid lines represent individual data points and the slopes of relationships, respectively. r and *p* indicate Pearson’s correlation coefficient and *p*-value. AN-R: restricting anorexia nervosa subtype; AN-BP: binging and purging anorexia nervosa subtype; SCD18: stearoyl-CoA desaturase-18 (oleic acid/stearic acid); D6D: delta-6-desaturase (gamma-linolenic acid/linoleic acid); D5D: delta-5-desaturase (arachidonic acid/dihomo-gamma-linolenic acid); ELOVL6: elongase-6 (stearic acid/palmitic acid); ELOVL2: elongase-2 (adrenic acid/arachidonic acid).

When stratified by AN subtype, n-3 ALA and DPA in AN-R were inversely correlated with BMI (r = −0.54, *p* = 0.01 and r = −0.53, *p* = 0.01). N-6 LA and GLA were positively correlated with BMI (r = 0.46, *p* = 0.02 and r = 0.48, *p* = 0.01), whereas n-9 mead acid was inversely correlated with BMI (r = −0.46, *p* = 0.02). In AN-BP, n-9 erucic acid was inversely correlated with BMI (r = −0.42, *p* = 0.04). Saturated margaric acid was inversely correlated with BMI in AN-R (r = −0.42, *p* = 0.04). In contrast, lauric acid and stearic acid were inversely correlated with BMI in AN-BP (r = −0.42, *p* = 0.02 and r = −0.48, *p* = 0.01). N-7 FAs were not significantly correlated with BMI in either subtype. Enzymes SCD18 and D6D were positively correlated with BMI in AN-R (r = 0.51, *p* = 0.01 and r = 0.40, *p* = 0.05), whereas none of the enzymes correlated with BMI in AN-BP (Figure 8A).

#### 2.7.2. Association with Age

N-6 eicosadienoic acid (20:2), DGLA, and saturated palmitic acid (16:0) were inversely correlated with age in all AN only (r = −0.30, *p* = 0.03; r = −0.37, *p* = 0.01; and r = −0.32, *p* = 0.02), whereas n-7 palmitoleic acid and n-9 nervonic acid (24:1 n-9) were inversely linked to age in controls only (r = −0.31, *p* = 0.04 and r = −0.35, *p* = 0.02). None of the n-3 PUFAs were significantly correlated with age in all AN or controls. Enzymes D5D and ELOVL2 were both positively correlated with age in all AN (r = 0.37, *p* = 0.01 and r = 0.34, *p* = 02), whereas SCD16 was inversely correlated with age in controls (r = −0.38, *p* = 0.01) (Figure 7B).

When stratified by AN subtype, n-6 adrenic acid (22:4) was positively correlated with age in AN-R (r = 0.46, *p* = 0.02), whereas DGLA was inversely correlated with age in AN-BP (r = −0.53, *p* = 0.01). Saturated myristic acid was inversely correlated with age in AN-R (r = −0.42, *p* = 0.03), while palmitic acid was inversely correlated with age in AN-BP (r = −0.40, *p* = 0.05). N-3, n-7, and n-9 FAs showed no significant correlations with age in either AN subtype. Enzyme D5D was positively correlated with age in AN-R (r = 0.44, *p* = 0.03), whereas none of the enzymes were correlated with age in AN-BP (Figure 8B).

#### 2.7.3. Associations with Psychiatric Phenotypes in AN

##### Different Patterns of Depression Association in AN Subtypes

In AN-R, every unit increase in n-3 11,14,17-eicosatrienoic acid (ETE, 20:3), and DPA was linked to an increased depression score by 0.01 (adjusted *p* = 0.03) and 0.0004 points (adjusted *p* = 0.04), respectively. Each unit increase in n-6 eicosadienoic acid and ARA was linked to a higher depression score by 0.004 points (adjusted *p* = 0.02) and 0.0001 points (adjusted *p* = 0.03). In AN-BP, every unit increase in eicosadienoic acid was linked to a decreased depression score by 0.004 points (adjusted *p* = 0.02). In contrast, each unit increase in DGLA was linked to an increased depression score by 0.002 points (adjusted *p* < 0.01). Every unit increase in n-9 godonic acid was associated with increased depression by 0.003 points (adjusted *p* = 0.03). In contrast, each unit increase in saturated pentadecanoic acid (15:0) and margaric acid was associated with an increased depression score by 0.002 points (adjusted *p* < 0.05) in AN-R only. None of the n-7 FAs and enzymes were associated with depression in either subtype.

##### Opposite Anxiety Association in AN Subtypes

**Figure 9:**
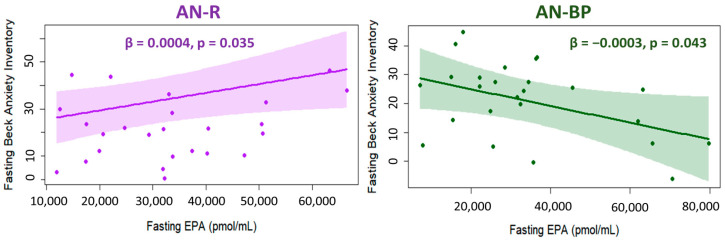
Association between fasting EPA and anxiety in restricting AN subtype (purple) and binging and purging AN subtype (green) after age, BMI, and assay batch adjustment. Colored dots, solid lines, and shaded areas represent individual residuals, slopes, and 95% confidence intervals. β and *p* indicate the beta-coefficient and *p*-value from multiple linear regression models. AN-R: restricting anorexia nervosa subtype; AN-BP: binging and purging anorexia nervosa subtype; EPA: eicosapentaenoic acid.

In AN-R, each unit increase in n-3 EPA and DPA led to a 0.0004-point increase in fasting timepoint anxiety (adjusted *p* = 0.04) and a 0.002% greater postprandial decrease in anxiety (adjusted *p* = 0.02). In AN-BP, each unit increase in EPA and ETE led to decreased fasting anxiety by 0.0003 points (adjusted *p* = 0.03) and 0.007 points (adjusted *p* = 0.02), respectively (Figure 9). 

In AN-R, every unit increase in n-6 osbond acid (22:5 n-6) and ARA was linked to 0.002 (adjusted *p* = 0.01) and 0.0001 point increase in anxiety score (adjusted *p* = 0.01), respectively. Each unit increase in eicosadienoic acid and DGLA was linked to 0.011% (adjusted *p* = 0.03) and 0.009% (adjusted *p* = 0.02) greater postprandial anxiety decrease. In comparison, every unit increase in n-9 mead acid and gadoleic acid (20:1 n-9) was linked to reduced postprandial anxiety by 0.006% (adjusted *p* = 0.01) and 0.01% (adjusted *p* < 0.01). Each unit increase in saturated lauric acid was associated with higher fasting anxiety by 0.0005 points (adjusted *p* = 0.04). In contrast, every unit increase in stearic acid was linked to a 0.0003% greater postprandial decrease in anxiety (adjusted *p* = 0.02). Each unit increase in enzyme ELOVL6 was associated with a 494% greater postprandial reduction in anxiety (adjusted *p* = 0.01). No significant relationships were identified in the AN-BP group.

##### High-Fat Food Aversion and Illness Duration Associations

None of the fasting FAs were significantly associated with high-fat food aversion in either subtype. In the enzyme associations, each unit increase in enzyme ELOVL2 was associated with a 1573% greater postprandial decrease in food aversion (adjusted *p* = 0.04) in AN-BP only. 

Higher n-3 EPA was associated with longer illness duration in AN-R only (adjusted *p* = 0.03), whereas higher n-6 GLA was associated with longer illness duration in AN-BP only (adjusted *p* = 0.02). The n-7, n-9, and saturated FAs were not significantly associated with illness duration in either subtype. Higher enzyme ELOVL5 was associated with shorter illness duration in AN-BP only (adjusted *p* = 0.02), whereas none of the enzymes were associated with illness duration in AN-R.

## 3. Discussion

Anorexia nervosa (AN) has recently been recognized as a “metabo-psychiatric disorder” [28]. Our work [24,29,49] has implicated metabolic dysregulation as a heritable risk factor underlying AN etiology. Genome-wide association studies and polygenic risk score findings have also identified genetic correlations between AN and metabolic phenotypes [27,28]. The gene we found associated with AN, *Epoxide Hydrolase 2* (*EPHX2*) [29], produces a critical regulatory enzyme (soluble epoxide hydrolase, sEH) in the metabolism of polyunsaturated n-3 and n-6 fatty acids (FAs) [29]. These results suggest that dietary lipids play an important role in a metabo-psychiatric disorder. While these data reinforced a genetic predisposition for lipid metabolic dysregulation in AN, it is unclear if disordered eating behavior further contributes to metabolic dysregulation in AN.

Lipid intake and composition can be affected by eating behaviors and dietary choices. Distinct disordered eating behaviors in the two AN subtypes enabled a deeper investigation into their impacts on lipid compositions. In general, AN patients are known to avoid high-calorie and high-fat foods [50] and prefer low-calorie and plant-based diets [17,51]. The two AN subtypes (AN-R and AN-BP) share a high aversion toward high-fat foods while otherwise engaging in distinct maladaptive eating behaviors to achieve and maintain pathological low weight. AN-R strictly restrict the amount and type of food consumed [30]. On the other hand, AN-BP are known to overeat in binging episodes and then purge using laxatives or self-induced vomiting [30], and they eat faster than AN-R [52]. Compared to controls, AN-BP are more preoccupied with thoughts of food, whereas AN-R have a lower urge to eat after a meal [52]. 

Eating behavior is clinically relevant, especially for individuals with AN. For example, higher binging frequency and preoccupation with thoughts of food commonly observed in AN-BP were both linked to a higher risk of depression [52]. AN-BP’s distinct eating behavior may contribute to findings of a higher burden of psychiatric comorbidities [38,39,40] and more severe symptomatology [35,36,37] compared to AN-R. While lipid dysregulation is now well-established in AN, little is known about how the aberrant lipids differ between the two subtypes. We utilized the differences in disordered eating behaviors of the two AN subtypes to explore how dietary behavior contributes to lipid dysregulation in AN. 

Adjusting for age, BMI, and assay batch, we found elevated n-3 (ALA, stearidonic acid, EPA, and DPA) and n-6 (LA and GLA) polyunsaturated fatty acids (PUFAs) in all AN compared to controls. These new results replicated our previous publications [24,25] and others’ works [20,21,22,23]. Conversely, other studies reported no significant differences [53] or lower n-3 and n-6 PUFA levels in their samples [21,23,54]. These studies tend to be more modest in sample size and have higher heterogeneity in the samples used. AN patients have been shown to prefer plant-based foods and fish while avoiding high-fat foods such as sausage [50]. Their dietary preferences may lead to increased n-3 PUFA intake and circulating levels, as n-3 PUFAs are obtained chiefly from fish, green leafy vegetables, or dietary supplementation [55,56]. Meanwhile, n-6 FAs are abundant in red meat and oils [57], which AN patients universally avoid. 

Genetic factors and lipid metabolism enzymes may also contribute to lipid dysregulation in AN. The expression of and variation in the genes encoding PUFA desaturases and elongases have been found to be associated with FA levels in healthy and psychiatric populations (bipolar disorder and depression) [58,59,60]. Two of these enzymes, D6D and ELOVL5, catalyze the first step of PUFA desaturation and elongation [61,62] and are altered in AN in this study. D6D was higher, whereas ELOVL5 was lower in AN-R and AB-BP compared to controls. The results of D6D and EOVL5 in AN are inconsistent in the literature; one study reported lower D6D in younger girls with eating disorders [53], while another found that adult women with AN showed no significant difference in D6D but higher ELOVL5 compared to controls [63]. While a lack of direct measurement of lipogenic enzymes and differences in study methodologies (specimens used, fasting/non-fasting conditions) contribute to these mixed findings, these results highlight the importance of age as a contributing factor to lipid metabolism in AN. 

Disordered eating behavioral differences between the two AN subtypes likely lead to additional metabolic perturbations. Compared to AN-R, AN-BP patients have been found to show more electrolyte abnormalities and gastrointestinal dysfunction, which can result in a more dynamic metabolic fluctuation of lipids [64]. Significant differences in maladaptive eating behaviors between the two AN subtypes may result in long-term differences in host metabolism. We found that ALA, stearidonic acid, EPA, DPA, LA, and GLA were significantly higher in AN-R compared to controls; however, only EPA was significantly higher in AN-BP. A Japanese study also reported different FA abnormalities between the two AN subtypes in their hospitalized patient samples before hospitalization, and also different FA recovery patterns after treatment completion [22]. These results suggest that subtypes’ differential disordered eating behavior and dietary intake can impact metabolism to promote distinct patterns of FA abnormalities.

Although n-3 EPA was elevated in both subtypes relative to controls, AN-R and AN-BP display different associations of EPA with anxiety despite having no differences in anxiety levels. Higher EPA was associated with higher anxiety in AN-R, whereas in AN-BP, higher EPA was associated with lower anxiety. AN-BP patients have been found to engage in more “anxiety eating” but less “happiness eating” than AN-R [65]. Excessive food intake in an anxious state can lead to FA elevation and temporary/partial alleviation of anxiety for AN-BP. In previous studies, n-3 PUFA (EPA, DPA, and DHA) intake has been linked to a lower risk of anxiety disorders [66], and n-3 supplementation was found to alleviate anxiety symptoms in various medical conditions, including depression [67]. However, a randomized controlled trial found no beneficial effects of EPA+DHA supplementation on anxiety in AN, but this trial enrolled only AN-R and not AN-BP patients [68]. It is unclear if the effect of n-3 PUFA supplementation on anxiety will reveal different results in AN-BP patients. Further research is needed to elucidate how AN subtypes differ in benefiting from n-3 or other PUFA supplementation to improve comorbidities such as anxiety.

Saturated fat is considered “unhealthy” and abundant in the modern Western diet. Saturated lauric acid can be found in coconut milk and oil, laurel oil, and palm kernel oil [69,70], which are commonly used in processed foods. Lauric acid was significantly higher in AN-BP compared to AN-R (magnitude of difference: 74%) and even to controls (43%). This elevation is likely attributed to AN-BP’s binge eating of processed foods. AN-BP have been shown to consume more high-fat, high-carbohydrate foods than AN-R [12], with pizza, bread, and pasta being common binge foods [71]. In addition, AN-BP frequently engage in “episodic dietary restraint” (i.e., skipping meals followed by binging and purging episodes), as opposed to AN-R’s “consistent dietary restraint” (i.e., eating regularly but with consistently limited amounts) [46]. Behavioral characteristics in AN-BP, such as high impulsivity [72,73], emotional dysregulation [72,73], and emotional eating [47,48] can also lead to repeated binging of high-fat processed foods, further contributing to the difficulty of controlling saturated fat intake. At our postprandial timepoint (two hours after the study meal), lauric acid increased significantly in AN-R yet unexpectedly decreased in AN-BP. This may reflect a compensatory mechanism to the baseline levels or an aberrant metabolic response from chronic exposure to a high-saturated-fat diet. The role of AN-BP’s disordered eating behavior in lipid dysregulation is supported by Shimizu et al. [22]. They found the normalization of lipid dysregulation differed between the two subtypes after 3 months of inpatient treatment—AN-BP’s normalization of monounsaturated FAs (MUFAs) was down to the levels of the healthy controls [22].

Lauric acid and other saturated and monounsaturated FAs have been linked to various metabolic and signaling pathways. For example, variation in one Cytochrome P450-pathway gene (*Cytochrome P450 Family 4 Subfamily V Member 2*, *CYP4V2*) has been linked to altered lauric acid oxidation [74]. Lauric acid has been shown to promote ketosis and display antibacterial, anti-inflammatory, and antioxidant properties [75,76]. In addition, lauric acid increases both the “bad” and “good” forms of cholesterol (low-density lipoprotein [LDL] and high-density lipoprotein [HDL]), raises total cholesterol, and improves the ratio of total cholesterol to HDL [66]. Coincidentally, hypercholesterolemia and elevated HDL and LDL are a hallmark of AN [77], which reinforces the clinical relevance of lipid dysregulation in AN pathogenesis. Saturated and monounsaturated FAs can also act as regulatory signal molecules in lipid and energy metabolism. For instance, peroxisome proliferator-activated receptor gamma coactivator 1-beta (PGC1-b), which is involved in FA beta-oxidation and energy expenditure regulation [78], is highly responsive to saturated palmitic and myristic acids [79]. Moreover, monounsaturated palmitoleic acid was termed the first lipokine as a whole-body fuel regulator [80]. These highlight the importance of paying closer attention to saturated and monounsaturated FAs in any human disorder involving aberrant lipid metabolism. 

A striking observation was the different lipid metabolic responses to our standardized study meal in AN versus controls. Two hours after eating, fifteen FAs and two lipogenic enzymes (D6D and ELOVL5) increased significantly in controls, whereas none of the FAs were significantly changed in AN. This is a novel finding supporting AN as a “metabo-psychiatric disorder” [28] that displays an abnormal response to diet. Delayed gastric emptying and intestinal transit in AN [81] may account partially for the lack of significant FA changes in AN; however, other GI medical comorbidities commonly present in AN [82] may also disrupt lipid uptake after eating. Notably, directionally opposite changes were observed between the two AN subtypes for lauric acid and ELOVL2 levels after eating. Subtype differences in dietary restraint [45], illness duration [83,84], eating disorder symptoms [35,36,37], and gastric emptying time [34] can all play a role in this finding. 

There may be differences in the normalization of PUFA dysregulation between AN subtypes. Our study design enabled us to compare PUFAs between each subtype’s ill AN and recovered AN groups. While recovered AN-BP displayed significantly different DHA, pentadecanoic acid, and total n-6/total n-3 ratio levels compared to ill AN-BP, none of the lipid markers were significantly different between ill and recovered AN in the AN-R group. Similarly, Shimizu et al. found subtype differences in FA changes after treatment. Six FAs (one saturated, one monounsaturated, two n-3, and two n-6) further increased in AN-R, whereas three FAs increased but two MUFAs normalized to the levels of the controls in AN-BP [22]. Differential FA changes observed between the two subtypes may reflect their differences in clinical trajectories and rates of recovery in metabolic, dietary, and psychosocial domains [34,45]. 

This study has several strengths. We confirmed and replicated previous findings, including data in our earlier studies, that FA dysregulation in AN was primarily driven by n-3 PUFA elevation. Moreover, our well-controlled meal exposure study protocol minimized variation and confounders to detect different responses to a high-fat meal between groups. This study was limited by the use of FA ratios to estimate enzyme activities. In addition, our AN group had a smaller sample size at the postprandial timepoint due to some participants refusing to eat the study meal. Nevertheless, sub-group analysis showed the findings to be consistent. This study analysis is based on well-known behavioral differences between the two AN subtypes. Our unpublished data confirmed this presumption that our AN-BP participants engage in more overeating and binging episodes, secret eating, vomiting, and laxative episodes compared to AN-R participants. Finally, it is important to note that the regional dietary habits of our participants, recruited from the USA and Canada, may limit the generalizability of our results globally.

AN is a deadly psychiatric disorder with limited treatment available. This work has underscored FA dysregulation as a reproducible hallmark of AN. We further demonstrated FA association with AN risk and phenotypes, and revealed distinct FA associations in the two AN subtypes. We identified significantly different meal-associated lipid changes in AN compared to healthy controls that support the “metabo-psychiatric disorder” characteristic of AN. Our data also clarified the different patterns of lipid dysregulation in the two AN subtypes at both fasting and postprandial timepoints, and showed differential PUFA associations with anxiety and depression in the two subtypes. FAs play an essential role in human health, and several polyunsaturated FAs are upstream sources of substrates for soluble epoxide hydrolase, an enzyme produced by an AN-associated gene. Thus, FAs serve as important biomarkers for neurobiological and psychiatric disorders such as AN. Given the limited treatment success for AN, future research and development of personalized nutritional interventions should be endorsed as adjunctive therapies to enhance treatment effectiveness. Advancing our knowledge of nutritional strategy can improve metabolic dysregulation and support recovery in AN. 

## 4. Materials and Methods

### 4.1. Participants and Study Design

Participants were recruited by the University of California-San Diego (UCSD) of California, USA, and the University of Toronto, Canada, from the university clinics, university campuses, and communities. Exclusion criteria included Axis I psychiatric illness, organic brain syndrome, schizophrenia or schizoaffective disorder, untreated thyroid disease, renal disease, hepatic disease, pregnancy or breastfeeding, and recent use of fish oil supplements. Participants with anorexia nervosa (AN) had to have a formal AN diagnosis by a clinician. The restricting (AN-R) and the binging and purging (AN-BP) subtypes were determined based on the Diagnostic and Statistical Manual of Mental Disorders [DSM]-V) [4]. As AN subtypes are behaviorally defined, our subtype groups comprise participants from all possible weight spectrums. Thus, we adjusted for BMI in our analyses to account for these weight variations. Ill AN was defined as having a BMI ≤ 17.5 kg/m^2^ and ongoing eating disorder symptoms, whereas recovered AN was defined as maintaining a satisfactory weight (BMI ≥ 18.5 kg/m^2^) with no eating disorder symptoms for at least one year.

A total of 50 women with AN (age: 30.18 ± 10.19) and 46 age-matched healthy control women (age: 30.67 ± 9.33) were enrolled. Blood samples and research questionnaires were collected from all participants after at least 10 h of fasting. All participants were asked to eat the study meal, which was a sausage, egg, and cheese muffin containing 436 kcalories, 27 g fat (11 g saturated FAs, 3.5 g n-6 PUFAs, 0.20 g n-3 PUFAs, n-6/n-3 ratio = 17.77:1), 28.5 g carbohydrates, and 19 g protein. All controls and 29 AN (58%) completed the study meal. Two hours after eating, blood samples and questionnaires were obtained again. This study has been approved by the University of Toronto Research Ethics Board and the UCSD Human Protection Board.

### 4.2. Phenotype Data

The Beck Depression Inventory (BDI)-II [85], Beck Anxiety Inventory (BAI) [86], and a Food Aversion Questionnaire (FAQ) were distributed to all participants at the study visit. Two hours after finishing the study meal, all participants completed the BAI and FAQ again. Higher questionnaire scores indicate higher phenotype severity.

### 4.3. Fatty Acid Analysis

Total plasma FA (sum of esterified and non-esterified) levels were measured by gas chromatography-mass spectrometry (GC-MS) at the UCSD Lipidomics Core (La Jolla, CA, USA), as previously reported [87]. To analyze the total plasma FA composition, the plasma lipids were first subjected to base-catalyzed hydrolysis. Human plasma (10 μL) in methanol (250 μL) was supplemented with a cocktail of internal standards consisting of 15 deuterated FAs, saponified with 0.5 N KOH for 30 min at 37 °C, and then adjusted to pH = 4 with glycine buffer. The FAs were extracted with isooctane and derivatized with pentafluorobenzyl (PFB) bromide. The resulting fatty acid PFB esters were analyzed by GC-MS using a negative chemical ionization mode (Agilent 6890N gas chromatograph equipped with an Agilent 5973 mass selective detector; Agilent, Santa Clara, CA, USA). All reagents and solvents were of the highest purity and suitable for mass spectral analyses. They were purchased from ThermoFisher Scientific (Waltham, MA, USA). All FA standards (purity > 99%) used for identification and quantification were purchased from Nu-Chek Prep Inc. (Elysian, MN, USA). All deuterated FAs used as internal standards were purchased from Cayman Chemical (Ann Arbor, MN, USA). Standard curves for each FA were acquired in parallel using identical conditions. The quantitative assessment of FAs in a sample was achieved by comparison of the mass spectrometric ion signal of the target molecule normalized to the internal standard with the matching standard curve according to the isotope dilution method. To ensure precision and accuracy, validation assays were conducted using composite unlabeled standards and deuterated internal standards as calibration controls, and FAs were analyzed in triplicate following routine procedures. FA levels are reported in pmol/mL.

### 4.4. Quality Control Protocol

We are aware that some saturated or monounsaturated FAs may be present as contaminants in our solvent system or even glassware. To compensate for potential contamination by exogenous FAs, we ran triplicate blank controls of deuterated internal standards parallel to the samples. These blank controls were processed using the same method as the samples and analyzed by GC. The absolute amount of contaminating FAs was calculated after normalization to the deuterated internal standards. The background contamination was then subtracted from the biological samples. Thus, the reported values represent the endogenous FA levels of the plasma.

Each FA was identified and annotated by its molecular mass and retention time, as previously reported [87]. For each of the individual FAs reported, we injected an identical commercially available FA standard and matched the retention time and molecular mass. We only reported FAs for which we have authentic standards and are confident that our annotations are accurate.

### 4.5. Fatty Acid Classes and Enzyme Activity Indexes

Individual FAs within the same class (saturated FAs, monounsaturated FAs [MUFAs], polyunsaturated FAs [PUFAs], n-3, n-6, n-7, and n-9) were summed as follows to quantify total FA levels: total saturated FAs: lauric acid + myristic acid + pentadecanoic acid + palmitic acid + margaric acid + stearic acid + arachidic acid; total MUFAs: palmitoleic acid + heptadecanoic acid + oleic acid + gadoleic acid + erucic acid + nervonic acid; total PUFAs: ALA + stearidonic acid + 11,14,17-eicosatrienoic acid (ETE) + EPA + DPA + DHA + LA + GLA + DGLA + ARA + adrenic acid + osbond acid + 5,8,11-eicosatrienoic acid; total n-3 PUFAs: ALA + stearidonic acid + ETE + EPA + DPA + DHA; total n-6 PUFAs: LA + GLA + DGLA + ARA + adrenic acid + osbond acid; total n-7 FAs: palmitoleic acid + heptadecanoic acid; total n-9 FAs: oleic acid + gadoleic acid + erucic acid + nervonic acid + 5,8,11-eicosatrienoic acid.

Ratios of product/substrate FAs were used as in vivo activity markers for the following desaturases and elongases: stearoyl-CoA desaturase-16 (SCD16): palmitoleic acid/palmitic acid [88]; stearoyl-CoA desaturase-18 (SCD18): oleic acid/stearic acid [53]; delta-6-desaturase (D6D): GLA/LA [88]; delta-5-desaturase (D5D): ARA/DGLA [88]; elongase-6 (ELOVL6): stearic acid/palmitic acid [89]; elongase-5 (ELOVL5): DGLA/GLA [89]; elongase-2 (ELOVL2): adrenic acid/ARA [90].

### 4.6. Statistical Analysis

Analysis of variance (ANOVA), Tukey post hoc tests, and Fisher’s exact tests were used to compare demographic and clinical characteristics between AN subtypes and for all AN or each AN subtype versus controls. Twenty-six FAs, seven FA classes, three FA ratios (total unsaturated FA [MUFA + PUFA]/total saturated FA, total PUFA/total saturated FA, total n-6/total n-3 PUFA), and seven enzyme activity indexes (three elongases and four desaturases) were compared between all AN and controls, and among three study groups (AN-R, AN-BP, and controls) at fasting and postprandial timepoints using analysis of covariance (ANCOVA) models adjusted for age, BMI, and assay batch. Fasting FAs were also compared between ill AN, recovered AN, and controls and between AN subtypes within each recovery status. Pearson’s correlations and covariate-adjusted linear regression models were used to assess FA and enzyme associations with AN-relevant phenotypes (age, BMI, depression, anxiety, high-fat food aversion, postprandial change in anxiety and food aversion, and illness duration). Statistical analysis was performed using R 4.3.1.

## Figures and Tables

**Table 1:** Study participant characteristics.

Characteristic	All AN (*n* = 50: IAN = 30; RecAN = 20)	AN-R (*n* = 25: IAN = 13; RecAN = 12)	AN-BP (*n* = 25: IAN = 17; RecAN = 8)	Controls (*n* = 46)
Age (years)	30.18 ± 10.19	27.56 ± 8.18	32.80 ± 11.44	30.67 ± 9.33
BMI (kg/m^2^)	18.92 ± 3.78 *	19.24 ± 4.38 *	18.64 ± 3.13 *	22.92 ± 3.52
Age of onset (years)	16.41 ± 4.26	15.78 ± 3.78	17.04 ± 4.70	NA
Duration of illness (years)	12.26 ± 8.93	10.09 ± 8.55	14.44 ± 8.95	NA
Beck Depression Inventory score	22.00 ± 16.59 *	20.08 ± 15.14 *	23.92 ± 18.03 *	4.00 ± 7.49
Fasting Beck Anxiety Inventory score	21.40 ± 13.69 *	21.64 ± 12.96 *	21.32 ± 14.65 *	4.33 ± 5.90
Postprandial change in Beck Anxiety Inventory score (%)	−10.91 ± 14.93 *	−36.03 ± 45.02 *	−47.17 ± 43.98 *	−10.98 ± 35.49
Fasting Food Aversion score	4.57 ± 2.76 *	4.63 ± 2.98 *	4.52 ± 2.57 *	1.94 ± 1.93
Postprandial change in Food Aversion score (%)	−6.97 ± 17.34 *	−7.87 ± 16.94	−6.00 ± 18.41	7.03 ± 30.75
Racial background, *n* (%)	White = 42 (84%) *; Asian = 7 (14%) *; African Americans = 0 (0%); Other = 1 (2%)	White = 21 (84%); Asian = 3 (12%); African Americans = 0 (0%); Other = 1 (4%)	White = 21 (84%); Asian = 4 (16%); African Americans = 0 (0%); Other = 0 (0%)	White = 26 (57%) Asian = 16 (35%); African Americans = 2 (4%); Other = 2 (4%)

Note: Data are in mean ± standard deviation. Comparisons between groups were tested using analysis of variance (ANOVA), Tukey post hoc tests, and Fisher’s exact tests. * = *p*-value < 0.05 comparing each anorexia nervosa (AN) group to controls. BMI: body mass index; IAN: ill anorexia nervosa; RecAN: recovered anorexia nervosa; AN-R: restricting anorexia nervosa subtype; AN-BP: binging and purging anorexia nervosa subtype; Other = more than one race.

## Data Availability

The data presented in this study are available on request from the corresponding author. The data are not publicly available at this time.

## Supplementary Materials

The following supporting information can be downloaded at https://www.mdpi.com/article/10.3390/ijms25105516/s1.
