# Supplementary material for: Fatty Acids and Their Lipogenic Enzymes in Anorexia Nervosa Clinical Subtypes
Source: Int J Mol Sci. 2024 May 18;25(10):5516. doi: 10.3390/ijms25105516 (PMC11122126; doi:10.3390/ijms25105516)
Supplement: Supplementary file 1 [file ijms-25-05516-s001.zip › ijms-2912307-supplementary.pdf]

Table S1: Fasting levels of fatty acids (pmol/mL), fatty acid ratios, and lipogenic enzyme activities in anorexia nervosa (AN) subtypes and healthy controls.

Marker	AN-R	AN-BP	Controls
Lauric acid (12:0)	8914.68 ± 9945.90	16830.55 ± 16701.84	10481.09 ± 4795.41
Myristic acid (14:0)	50136.54 ± 30694.17	73790.11 ± 93474.3	50916.08 ± 21685.32
Pentadecanoic (15:0)	11288.57 ± 4482.27	10378.23 ± 4694.72	10599.37 ± 5244.86
Palmitic acid (16:0)	1005743.20 ± 206127.37	1070063.39 ± 263743.18	1025960.57 ± 344304.59
Palmitoleic acid (16:1)	57936.60 ± 44632.79	96888.91 ± 128445.35	68043.53 ± 29452.17
Margaric acid (17:0)	14534.13 ± 4003.11	13055.27 ± 4512.53	13876.79 ± 6315.98
Heptadecanoic acid (17:1)	6028.88 ± 2866.68	6480.07 ± 3818.77	6362.45 ± 3181.18
Stearic acid (18:0)	305166.43 ± 85513.97	328309.94 ± 66781.38	323459.30 ± 130196.54
Oleic acid (18:1)	27123.58 ± 4685.16	28421.42 ± 9204.47	24898.3 ± 4921.32
Linoleic acid (LA, 18:2)	59263.21 ± 11112.09	60759.18 ± 15933.03	53759.62 ± 14851.29
Alpha-linolenic acid (ALA, 18:3 n-3)	1967.63 ± 823.35	1720.28 ± 1753.45	1438.60 ± 505.45
Gamma-linolenic acid (GLA, 18:3 n-6)	1978.85 ± 897.01	1674.55 ± 468.71	1434.15 ± 930.74
Stearidonic acid (18:4)	89.14 ± 57.03	74.06 ± 78.6	55.77 ± 44.18
Gadoleic acid (20:1)	5085.97 ± 28	6760.94 ± 6422.71	6251.28 ± 4997.82
Eicosadienoic acid (20:2)	4851.88 ± 1832.92	4955.08 ± 2007.16	5357.85 ± 2744.99
11,14,17-eicosatrienoic acid (ETE, 20:3 n-3)	1337.83 ± 546.94	1252.33 ± 973.67	1321.15 ± 942.57
Dihomo-gamma-linolenic acid (DGLA, 20:3 n-6)	70084.85 ± 23843.19	72807.93 ± 23463.11	67504.50 ± 35614.33
5,8,11-eicosatrienoic acid (20:3 n-9)	4636.02 ± 4847.54	5231.92 ± 5519.49	4419.48 ± 3809.78
Arachidonic acid (ARA, 20:4)	311016.06 ± 85338.71	329178.15 ± 105018.75	323960.90 ± 124173.96
Eicosapentaenoic acid (EPA, 20:5)	31166.98 ± 14066.38	32264.07 ± 20645.57	24117.29 ± 10821.17
Erucic acid (22:1)	124.24 ± 267.7	106.74 ± 85.19	115.90 ± 70.96
Adrenic acid (22:4)	7316.02 ± 3385.61	8227.12 ± 3567.16	8435.35 ± 3913.56
Docosapentaenoic acid (DPA, 22:5 n-3)	30390.87 ± 14342.64	27020.93 ± 12267.54	24527.07 ± 10091.11

Osbond acid (22:5 n-6)	15598.74 ± 4130.66	18633.54 ± 11097.56	17540.27 ± 8846.25
Docosahexaenoic acid (DHA, 22:6)	128916.27 ± 54949.75	142936.61 ± 74238.23	147660.48 ± 71480.59
Nervonic acid (24:1)	148.38 ± 97.02	164.63 ± 98.83	152.07 ± 104.27
Total saturated fatty acids (SFA)	1395783.55 ± 307898.67	1512427.49 ± 401361.78	1435293.21 ± 490248.97
Total monounsaturated fatty acids (MUFA)	96447.65 ± 49825.06	138822.70 ± 133259.76	105823.53 ± 37917.36
Total polyunsaturated fatty acids (PUFA)	668614.35 ± 136291.54	706735.74 ± 195819.72	681532.47 ± 239452.54
Total n-3 PUFA	193868.72 ± 55985.04	205268.28 ± 99263.8	199120.35 ± 83982.42
Total n-6 PUFA	470109.61 ± 105605.21	496235.54 ± 128137.38	477992.64 ± 166474.50
Total n-7 fatty acids	63965.47 ± 47006.08	103368.98 ± 131468.66	74405.98 ± 32278.60
Total n-9 fatty acids	37118.20 ± 7542.74	40685.64 ± 14477.76	35837.03 ± 8809.98
Total PUFA/total SFA	0.49 ± 0.10	0.47 ± 0.05	0.49 ± 0.08
Total unsaturated (MUFA+PUFA)/total SFA	0.57 ± 0.10	0.56 ± 0.06	0.56 ± 0.09
Total n-6/total n-3 PUFA	2.65 ± 0.67	2.8 ± 0.99	2.65 ± 0.78
Stearoyl-CoA desaturase-16 (SCD16)	0.06 ± 0.04	0.08 ± 0.07	0.06 ± 0.02
Stearoyl-CoA desaturase-18 (SCD18)	0.11 ± 0.02	0.1 ± 0.04	0.09 ± 0.07
Delta-6-desaturase (D6D)	0.03 ± 0.01	0.02 ± 0.01	0.02 ± 0.01
Delta-5-desaturase (D5D)	4.94 ± 1.88	4.94 ± 1.88	5.46 ± 1.88
Elongase-6 (ELOVL6)	0.3 ± 0.05	0.31 ± 0.05	0.31 ± 0.06
Elongase-5 (ELOVL5)	198.76 ± 130.05	216.24 ± 196.42	295.94 ± 257.58
Elongase-2 (ELOVL2)	0.02 ± 0.01	0.03 ± 0.01	0.03 ± 0.01

Note: Data are in mean ± standard deviation. AN-R: restricting anorexia nervosa subtype; AN-BP: binging and purging anorexia nervosa subtype; total SFA: lauric acid + myristic acid + pentadecanoic acid + palmitic acid + margaric acid + stearic acid + arachidic acid; total MUFA: palmitoleic acid + heptadecanoic acid + oleic acid + gadoleic acid + erucic acid + nervonic acid; total PUFA: ALA + stearidonic acid + ETE + EPA +

DPA + DHA + LA + GLA + DGLA + ARA + adrenic acid + osbond acid + 5,8,11-eicosatrienoic acid; total n-3 PUFA: ALA + stearidonic acid + ETE + EPA + DPA + DHA; total n-6 PUFA: LA + GLA + DGLA + ARA + adrenic acid + osbond acid; total n-7 fatty acids: palmitoleic acid + heptadecanoic acid; total n-9 fatty acids: oleic acid + gadoleic acid + erucic acid + nervonic acid + 5,8,11-eicosatrienoic acid; SCD16: palmitoleic acid/palmitic acid; SCD18: oleic acid/stearic acid; D6D: GLA/LA; D5D: ARA/DGLA; ELOVL6: stearic acid/palmitic acid; ELOVL5: DGLA/GLA; ELOVL2: adrenic acid/ARA.

Table S2: Postprandial levels of fatty acids (pmol/mL), fatty acid ratios, and lipogenic enzyme activities in anorexia nervosa (AN) subtypes and healthy controls.

Marker	AN-R	AN-BP	Controls
Lauric acid (12:0)	10813.00 ± 6287.88	12571.02 ± 12246.17	9261.42 ± 5077.94
Myristic acid (14:0)	57184.72 ± 32951.15	70150.33 ± 87367.68	49634.63 ± 23688.96
Pentadecanoic (15:0)	11652.94 ± 3598.73	10489.22 ± 4533.44	10665.83 ± 5262.18
Palmitic acid (16:0)	1041851.57 ± 165265.53	1085918.50 ± 335867.13	1039544.15 ± 350885.39
Palmitoleic acid (16:1)	64642.12 ± 34507.2	86754.79 ± 137262.86	61011.34 ± 31089.03
Margaric acid (17:0)	14038.18 ± 3880.24	13059.39 ± 5284.44	14040.13 ± 6571.54
Heptadecanoic acid (17:1)	6328.83 ± 2702.40	5605.26 ± 2871.74	6487.6 ± 3905.22
Stearic acid (18:0)	307031.03 ± 76442.75	316064.00 ± 113132.50	326863.17 ± 141596.56
Oleic acid (18:1)	28138.57 ± 4857.29	24390.16 ± 4235.2	25165.75 ± 4975.84
Linoleic acid (LA, 18:2)	63628.73 ± 10969.57	65914.61 ± 18745.1	58010.31 ± 15986.77
Alpha-linolenic acid (ALA, 18:3 n-3)	1984.82 ± 759.27	1554.55 ± 651.19	1232.71 ± 468.15
Gamma-linolenic acid (GLA, 18:3 n-6)	2194.88 ± 1030.47	2181.60 ± 2338.22	1631.15 ± 1146.34
Stearidonic acid (18:4)	85.29 ± 50	70.50 ± 59.49	55.72 ± 45.26
Gadoleic acid (20:1)	4807.98 ± 2744.39	5766.00 ± 4158.26	6600.83 ± 5282.75
Eicosadienoic acid (20:2)	4598.27 ± 1662.59	4664.74 ± 1770.23	5397.35 ± 2996.83
11,14,17-eicosatrienoic acid (ETE, 20:3 n-3)	1268.20 ± 555.38	1083.14 ± 743.46	1348.20 ± 1056.8
Dihomo-gamma-linolenic acid (DGLA, 20:3 n-6)	71763.62 ± 25950.23	68845.15 ± 24901.50	70845.70 ± 39445.71
5,8,11-eicosatrienoic acid (20:3 n-9)	3689.31 ± 4337.47	5778.44 ± 6218.61	4168.36 ± 3736.23
Arachidonic acid (ARA, 20:4)	319198.42 ± 77896.35	351298.38 ± 139348.71	343075.11 ± 133327.06
Eicosapentaenoic acid (EPA, 20:5)	29411.89 ± 18024.86	40029.01 ± 26408.09	24266.61 ± 11398.68
Erucic acid (22:1)	107.88 ± 116.17	86.44 ± 56.24	100.35 ± 56.28
Adrenic acid (22:4)	6710.72 ± 2062.1	7966.78 ± 4055.25	8691.34 ± 4393.75
Docosapentaenoic acid (DPA, 22:5 n-3)	26405.93 ± 11006.72	28928.20 ± 10530.21	23597.35 ± 9700.56

Osbond acid (22:5 n-6)	17247.99 ± 6144.73	19782.89 ± 14426.29	18858.13 ± 10238.02
Docosahexaenoic acid (DHA, 22:6)	153216.56 ± 60386.01	170090.73 ± 93032.17	158394.28 ± 68245.39
Nervonic acid (24:1)	124.21 ± 102.72	143.67 ± 129.44	146.01 ± 118.22
Total saturated fatty acids (SFA)	1442571.45 ± 232508.39	1508252.46 ± 496761.19	1450009.32 ± 505674.96
Total monounsaturated fatty acids (MUFA)	104149.59 ± 41867.91	122746.32 ± 141730.26	99511.87 ± 40956.83
Total polyunsaturated fatty acids (PUFA)	701404.61 ± 160067.23	768188.71 ± 270699.8	719572.33 ± 249414.5
Total n-3 PUFA	212372.67 ± 80320.76	241756.13 ± 120926.75	208894.88 ± 81156.91
Total n-6 PUFA	485342.63 ± 99564.9	520654.15 ± 179306.35	506509.09 ± 179432.49
Total n-7 fatty acids	70970.95 ± 36906.64	92360.04 ± 139781.97	67498.93 ± 34495.4
Total n-9 fatty acids	36867.96 ± 7761.81	36164.71 ± 9230.07	36181.30 ± 9486.03
Total PUFA/total SFA	0.49 ± 0.08	0.51 ± 0.07	0.50 ± 0.08
Total unsaturated (MUFA+PUFA)/total SFA	0.56 ± 0.07	0.59 ± 0.08	0.57 ± 0.08
Total n-6/total n-3 PUFA	2.54 ± 0.66	2.47 ± 0.88	2.62 ± 0.63
Stearoyl-CoA desaturase-16 (SCD16)	0.06 ± 0.02	0.07 ± 0.07	0.06 ± 0.02
Stearoyl-CoA desaturase-18 (SCD18)	0.11 ± 0.03	0.10 ± 0.09	0.10 ± 0.07
Delta-6-desaturase (D6D)	0.03 ± 0.01	0.02 ± 0.02	0.02 ± 0.01
Delta-5-desaturase (D5D)	5.12 ± 2.09	5.29 ± 1.75	5.48 ± 1.72
Elongase-6 (ELOVL6)	0.29 ± 0.06	0.29 ± 0.08	0.31 ± 0.08
Elongase-5 (ELOVL5)	178.22 ± 143.36	148.15 ± 62.7	252.46 ± 194.76
Elongase-2 (ELOVL2)	0.02 ± 0.01	0.02 ± 0.01	0.03 ± 0.01

Note: Data are in mean ± standard deviation. AN-R: restricting anorexia nervosa subtype; AN-BP: binging and purging anorexia nervosa sub-type; total SFA: lauric acid + myristic acid + pentadecanoic acid + palmitic acid + margaric acid + stearic acid + arachidic acid; total MUFA: palmitoleic acid + heptadecanoic acid + oleic acid + gadoleic acid + erucic acid + nervonic acid; total PUFA: ALA + stearidonic acid + ETE + EPA + DPA + DHA + LA + GLA + DGLA + ARA + adrenic acid + osbond acid + 5,8,11-eicosatrienoic acid; total n-3 PUFA: ALA + stearidonic acid + ETE + EPA + DPA + DHA; total n-6 PUFA: LA + GLA + DGLA + ARA + adrenic acid + osbond acid; total n-7 fatty acids: palmitoleic acid + heptadecanoic acid; total n-9 fatty acids: oleic acid + gadoleic acid + erucic acid + nervonic acid + 5,8,11-eicosatrienoic acid; SCD16: palmitoleic acid/palmitic acid; SCD18: oleic acid/stearic acid; D6D: GLA/LA; D5D: ARA/DGLA; ELOVL6: stearic acid/palmitic acid; ELOVL5: DGLA/GLA; ELOVL2: adrenic acid/ARA.

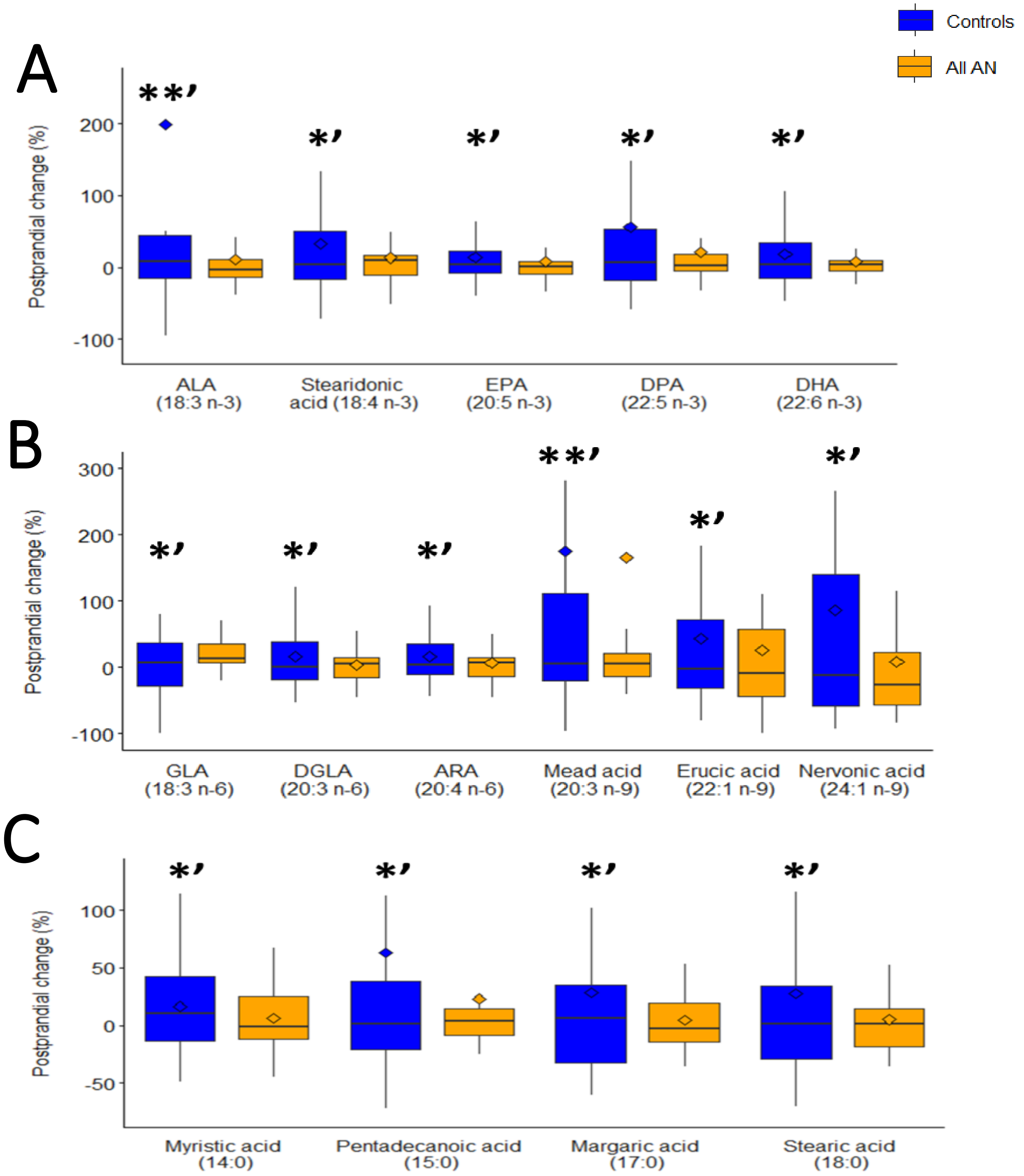

Figure S1: Fatty acids that showed significant postprandial percent changes in controls (blue) but not in AN (orange). n-3 (**A**), n-6 and n-9 (**B**), and saturated (**C**) fatty acids are presented in panels **A-C**. Boxplots indicate the first quartile, median, and third quartile. Diamond shapes represent the mean percent change. *′ and **′ represent $0.01 \le p < 0.05$ and $p < 0.01$, respectively, using one-sample t-tests. AN: anorexia nervosa; ALA: alpha-linolenic acid; EPA: eicosapentaenoic acid; DPA: docosapentaenoic acid;

DHA: docosahexaenoic acid; GLA: gamma-linolenic acid; DGLA: dihomo-gamma-linolenic acid; ARA: arachidonic acid.
